# Pleurisy as a Sign of Chronic Lymphocytic Leukemia

**DOI:** 10.7759/cureus.36880

**Published:** 2023-03-29

**Authors:** Afaf Thouil, Mohamed Lakhal, Houda Bachir, Amal Bennani, Hatim Kouismi

**Affiliations:** 1 Department of Respiratory Diseases, Laboratory of Research and Medical Sciences, Mohammed VI University Hospital, Faculty of Medicine and Pharmacy of Oujda, Mohammed First University, Oujda, MAR; 2 Department of Internal Medicine, Laboratory of Immunohematology and Cellular Therapy, Faculty of Medicine and Pharmacy of Oujda, Mohammed First University, Oujda, MAR; 3 Department of Anatomopathology, Faculty of Medicine and Pharmacy of Oujda, Mohammed First University, Oujda, MAR; 4 Department of Pulmonology, Mohammed VI University Hospital, Oujda, MAR

**Keywords:** malignant blood diseases, chemotherapy, leukemic effusion, pleurisy, the diagnosis of lymphoid leukemia (cll)

## Abstract

The diagnosis of chronic lymphoid leukemia (CLL) is essentially based on a blood smear and immunophenotyping by flow cytometry of circulating lymphocytes. Unusual locations of the disease can sometimes be observed. Here we report the case of a patient admitted for the management of pleurisy. The pleural effusion was lymphocytic exudate; histological examination of the pleural biopsy along with immunohistochemistry helped yield the diagnosis of secondary localization of CLL. The patient was transferred to the Internal Medicine department where chemotherapy was introduced.

## Introduction

Chronic lymphocytic leukemia (CLL) is the most common leukemia in adults and accounts for 12% of all blood diseases. This is a hematology with a heterogeneous clinical course: a third of the patients never require treatment, a third are symptomatic and require treatment right away, and the last third are treated during follow-ups. This heterogeneity is related to the characteristics of the tumor cell, especially molecular [1].

## Case presentation

**Figure 1:**
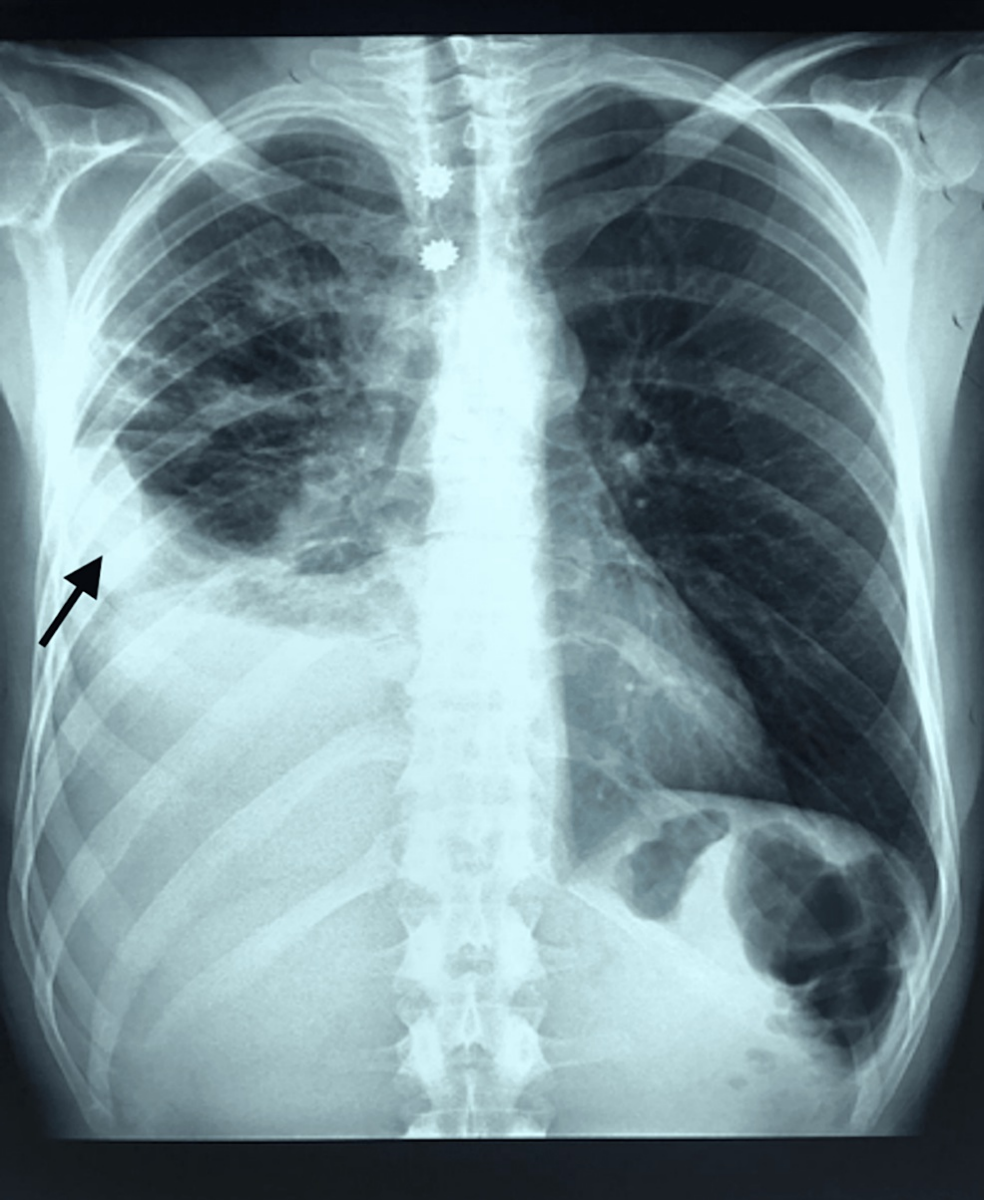
Chest X-ray showing right unilateral pleural effusion (black arrow)

A 55-year-old man, with no previous medical history, presented to the hospital with sharp left-sided chest pain and dry cough since three days. On clinical examination, the patient was found to be hemodynamically stable; oxygen saturation measured by pulse oximetry was 94% on room air. His chest X-ray showed right unilateral pleural effusion (Figure 1).

**Figure 2:**
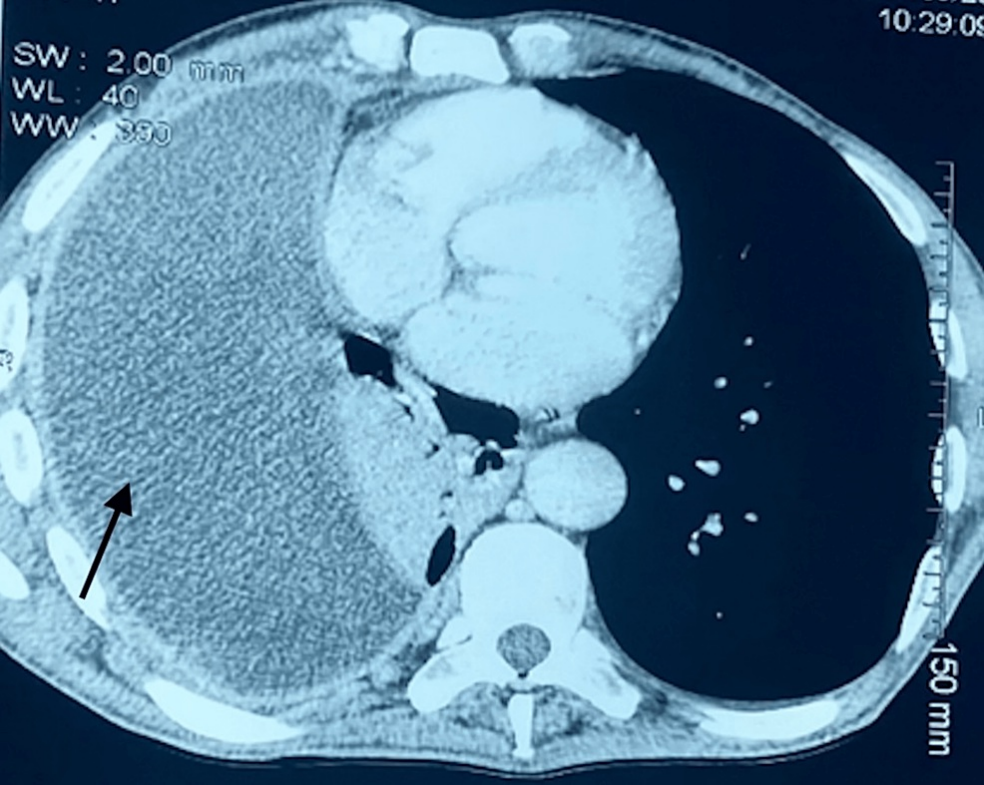
A chest-abdominal CT scan showing right profuse pleurisy without mediastinal adenopathy (black arrow)

On thoracocentesis, the liquid was amber and exudate with a predominance of lymphocytes. Cultures for *Mycobacterium tuberculosis* were negative, and no atypical cells were found in cytological analysis. A chest-abdominal CT scan showed moderate- to high-abundance pleurisy with no evidence of mediastinal lymphadenopathy, nor hepatomegaly or splenomegaly (Figure 2).

The diagnostic workup showed hyperleukocytosis at 116,250 cells/mm^3^ and lymphocytosis at 100,070 cells/mm^3^ without anemia or thrombocytopenia or other abnormalities. Immunophenotyping showed a monotypic B cell population with CD19+, CD5+, CD23+, CD43+, FMC7-, CD79b-, and CD20+ (low intensity), expressing a low-intensity kappa light chain. The Matutes score was calculated as 5. Overall, the phenotypic appearance was in favour of CLL.

**Figure 3:**
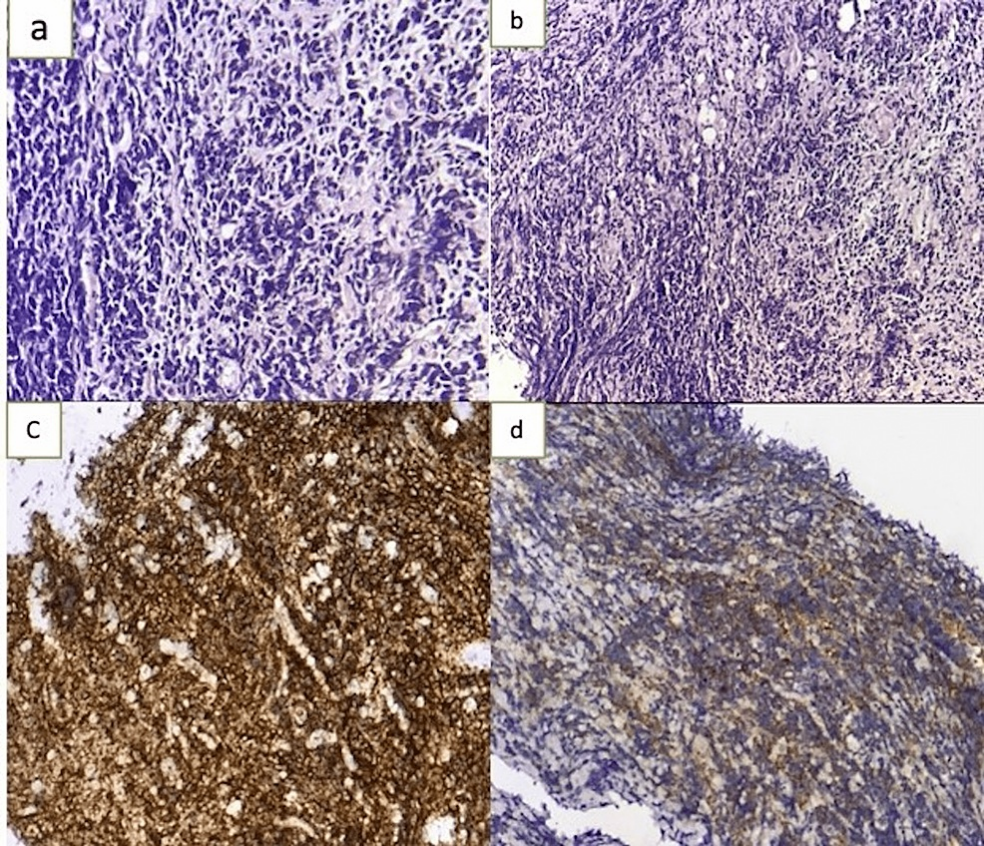
Histological examination of the specimen showed a diffuse proliferation of small, discohesive, monomorphic cells with little cytoplasm and nuclei with condensed chromatin, H&E x40-100 (a, b). Immunohistochemical examination showed diffuse and strong positivity for CD20 in the tumour cells (c), and weak positivity for CD23 (d).

A blind pleural biopsy was performed. The histological study showed a diffuse tumour proliferation, made up of small, lymphoid-looking, non-cohesive, squashed tumour cells, arranged in a sheet and with irregular, moderately pleomorphic nuclei (Figure 3). Immunohistochemistry showed positive labelling of tumour cells by CD20, CD5, CD23 and BCL2, while the labelling of tumour cells by CD10, CD3, and cyclin D1 was negative. A morphologic and immunohistochemical appearance of poorly differentiated tumour proliferation is primarily suggestive of chronic lymphocytic leukemia.

The diagnosis of Binet stage B chronic lymphocytic leukemia was made. The patient was transferred to the Internal Medicine department and was treated with a protocol of fludarabine, cyclophosphamide and rituximab (FCR). He is currently on his third course of treatment.

## Discussion

Of all adult hematological malignancies, chronic lymphocytic leukemia is the most common. Its clinical course is considered to be heterogeneous, and hence, the initiation of treatment varies according to its evolution. This heterogeneity in the behavior of the tumour cell is explained by several characteristics, notably molecular [1,2]. Apart from mediastinal adenopathy, thoracic manifestations of CLL are uncommon, including pulmonary infiltrates and pleural effusions. However, cases of CLL with pleurisy are very rare in the literature [3,4]. Despite its rarity, leukemic pleural involvement can occur even in the early stages of CLL. Pleurisy is often due to excretion of malignant cells and lymphatic obstruction as a result of lymphomatous infiltration of the pulmonary and mediastinal lymph nodes or thoracic duct [5].

In our setting, CLL manifested only as pleural effusion without evidence of mediastinal involvement; hence, our case is rare. The differential diagnosis in patients with CLL is infection, primary pleural involvement, central lymphatic obstruction, pleural infiltration by other neoplasia and changes induced by previous irradiation or chemotherapy [4,6,7]. There are several cytological, histological and immunological methods that can be used to determine the etiology of pleural effusion [4,7,8]. In pleural effusions due to CLL infiltration, the lymphocytes are predominantly B cells, whereas in pleural effusions due to other causes such as tuberculosis and pulmonary emboli, the lymphocytes are predominantly T cells [4]. The true incidence of leukemic effusion is not known. It may occur at presentation, during advanced, refractory disease or during a relapse [9-11]. The prognosis is variable, but remains poor in most cases. Treatment priorities include chemotherapy with fludarabine, cyclophosphamide and rituximab, or ibrutinib or bendamustine with or without CD20 monoclonal antibody [5]. Successful treatment has been reported with either induction chemotherapy or stem cell transplantation [9,12].

## Conclusions

Chronic lymphocytic leukemia is the most frequent adult leukemia. The diagnosis is based on the blood smear examination and immunophenotyping by flow cytometry of blood lymphocytes. Leukemic involvement of the pleura is uncommon but may occur even in an early stage of CLL, in the absence of more common causes or mediastinal abnormalities. Successful treatment is seen with either induction chemotherapy or stem cell transplant.
